# Damage Location Monitoring of Graphene/Conducting Polymer Composites Film Based on Self-Sensing

**DOI:** 10.3390/nano12162823

**Published:** 2022-08-17

**Authors:** Huihui Guo, Yuhang Li, Tingting Liu, Zuquan Wu

**Affiliations:** 1 School of Information Engineering, Southwest University of Science and Technology, Mianyang 621010, China; 2 Robot Technology Used for Special Environment Key Laboratory of Sichuan Province, Mianyang 621010, China; 3 School of Electrical Engineering and Electronic Information, Xihua University, Chengdu 611743, China

**Keywords:** conducting polymer composites, graphene composites, defect self-monitoring, sensing film, damage location

## Abstract

Conductive graphene polymer composites are considered promising functional materials in gas detection, strain detection, metal corrosion prevention, and electromagnetic wave absorption, owing to their good flexibility, lightweight, and adjustable conductivity. The internal defects or external damages of composite films will seriously affect the electrical and functional properties of the materials. Based on the conductive network inside the conductive polymer film and the self-inductance to ultrasonic wave, the defect self-monitoring system of the conductive polymer film is designed and optimized in this work. The self-damage detection system is composed of an electrode array, excitation source, resistance signal acquisition and processing circuit, and damage display. Aiming at different scenarios, the improved interdigital structure transducer for sensors and damage detection device for coating film with a large area are presented and optimized respectively. Meanwhile, the damage location algorithm based on time difference measurement and kernel density estimation algorithm is also optimized. The multiple damage detection is realized by a device with a 4 × 8 electrode array, and the relative error of damage area with 1 mm × 1 mm is less than 5%, and the lower detection limits of damage size are 0.3 mm × 0.3 mm.

## 1. Introduction

Conducting polymer is a unique organic semiconductor functional material with abundant properties in electronics, optics, and magnetism, which are widely used in the fields of aerospace, military, transportation, sports apparatus, and electronic devices [1,2,3,4]. Graphene is a typical 2D material with exciting fascinating features such as the highest strength and Young’s modulus ever measured, gigantic specific surface area, best-known thermal conductivity, and electrical conductivity, which is an ideal filler for high-performance multifunctional nanocomposites in light of its superior mechanical, electrical, thermal, and optical properties [5]. Graphene polymer composites with high performance are applied in sensors [6], actuators [7,8], energy storage [9], and absorbing and protective coating [10,11]. Trajcheva, A. et al. [6] proposed a waterborne polymer/Graphene Nanoribbon nanocomposites for low concentrations of toxic gases detection (CO, NH_3_, and N_2_O in a concentration range of 70–1000 ppm), it was found that the sensors are characterized by a large sensor response in a short time, at room temperature and with very good reproducibility in three investigated cycles of gas adsorption and desorption; Panahi-Sarmad, M. et al. [7] reviewed the latest progress and potential application scenarios of graphene actuators; James Loomis et al. [8] prepared nanoplatelet/polydimethylsiloxane (PDMS) composites as photomechanical actuators and studied their infrared (IR) mechanical responses with increasing pre-strains; Li et al. [11] prepared polyaniline modified graphene nanocomposites as anti-corrosion coatings and showed good anti-corrosion effects.

The performance of graphene/polymer nanocomposites depends on graphene configuration, distribution in polymer, and interfacial interaction between graphene and polymer [12]. The distribution condition of filling particles in polymer and the internal damage defects of polymer composites is determined by the fabrication process. The preparation methods of polymer composites mainly include a conductive material filling method and a blending method. In particular, the filling method is easy to produce pores in the film-forming process, which will seriously affect the performance of the film [13]. In addition, the structural damage of the composite films also affects the properties of the films used as a protective coating [11] and a microwave-absorbing coating [14]. In aerospace and military applications, it is of great significance to detect the structural health of those films and the rapid location of structural damage in films.

Generally, the commonly used damage detection methods include the ray method, thermal imaging method, and ultrasonic method. X-ray and thermal imaging methods not only need expensive instruments and equipment but also may change the characteristics of functional films. The ultrasonic method is suitable for non-destructive testing of graphene composite films. However, the ultrasonic sensors must be externally connected or internally embedded in composite materials [15].

In recent years, the nanocomposite film with the capability of self-perceiving guided ultrasonic waves has been reported [16]. For graphene/polymer films, the surpassing electrical property of graphene could improve the electrical conductivity of polymers by building a conductive network for free electrons. The conductive mechanism includes a conductive channel, tunneling effect, and field emission [17]. When the content of graphene is small, the tunneling effect is dominant. At the same time, the graphene is formed into a network, in which the quantum tunneling effect can be locally triggered when guided ultrasonic waves traverse the composites. The diffuse sensing network makes it possible to acquire guided ultrasonic waves at any point of the polymer, avoiding ultrasonic sensors internally embedded in the composites. Subsequently, Ye, L. et al. [18] proposed the “sensor-free” structural health monitoring initiative using graphene-functionalized as sensing film for glass fiber-reinforced composites, which has spotlighted a new breed of functional polymers with the capability of self-health monitoring. However, the device with 300 mm × 300 mm × 1mm is too large, which is only suitable for the detection of large-area plates, and the accuracy of damage location is not high enough to meet the small defects detecting.

**Figure 1:**
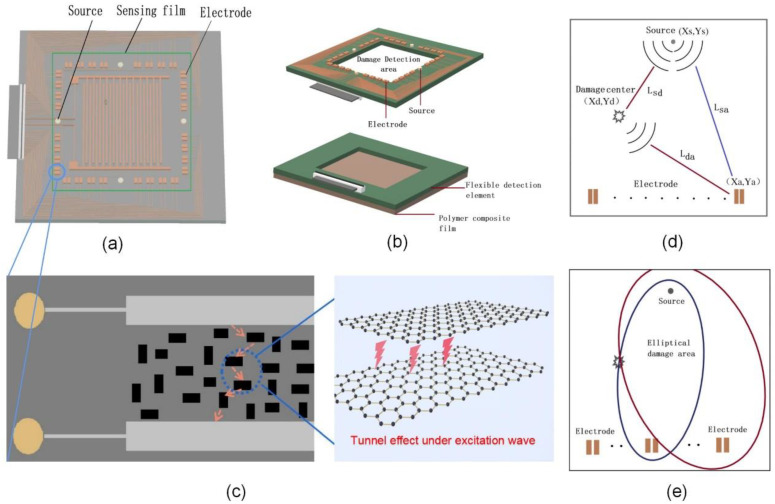
(**a**) The transducer of the sensor with electrode array and excitation source; (**b**) the flexible device of health monitoring for the composite polymer; (**c**) Tunneling effect of conducting polymer under excitation wave; (**d**) Schematic of wave propagation inside the film; (**e**) Schematic of ellipse image based on triangulation.

Due to the conductive network inside the conductive polymer film and the self-inductance to ultrasonic waves, the defect self-monitoring system of the conductive polymer film is designed and optimized in this work. Aiming at the sensor application field, the detection area is set to 30 mm × 30 mm, and the interdigital transducer with integrated electrode array and excitation source using microelectronic technology is presented as shown in Figure 1a. For the application of coating film health monitoring, the structure of a flexible device is also proposed as shown in Figure 1b. In addition, the damage location algorithm based on time difference measurement is also optimized to improve the positioning accuracy of multiple damage locations.

## 2. Self-Monitoring Method and Principle

The 2-D graphene nanoparticles as conductive nano-fillers are evenly dispersed in a dielectric polymer (such as epoxy resin, polyimide, polyethylene, polyurethane, glass fiber, silicone rubber, polyaniline, etc), which can create an electrical network in the polymer. The conductive mechanism includes a conductive channel, tunneling effect, and field emission. Generally, the nano-fillers content is closely related to the conductive mechanism of polymers. The tunneling effect is particularly prominent for nanocomposites film when two nanoparticles are nearby but not in direct contact, and the quantum tunneling effect dominates the electrical resistance manifested by the nanocomposites, which brings somewhat appealing and unique properties to nanocomposites such as semi-conductive properties.

It is well-known that ultrasonic is widely used in structural flaw detection owing to high sensitivity to structural damage, omnidirectional dissemination, fast propagation, and strong penetration through waveguide thickness. As elastic waves traverse conducting polymer, the wave-induced strain modulates the inter-distance among nanoparticles and consequently triggers the quantum tunneling effect, which alternates the measured resistivity of the polymer as shown in Figure 1c. Due to the self-responding to the ultrasonic wave, the moment when the ultrasonic wave passes through can be obtained by detecting the resistivity transformation at the same position.

The same excitation wave will be scattered by defects in the polymer and reach the same position through different paths as shown in Figure 1d. The time difference between the scattering signal of wave damage and the excitation signal reaching each electrode array is extracted by the time-of-flight method, and the location of damage (*x_d_*, *y_d_*) at any in the inspected composite structure can be triangulated as [19].

(1) $$\begin{array}{l} \Delta t=(\frac{L_{sd}}{V_{1}}+\frac{L_{da}}{V_{2}})-\frac{L_{sa}}{V_{1}}\\ L_{sd}=\sqrt{{\left(x_{d}-x_{s}\right)}^{2}+{\left(y_{s}-y_{d}\right)}^{2}}\\ L_{da}=\sqrt{{\left(x_{a}-x_{d}\right)}^{2}+{\left(y_{d}-y_{a}\right)}^{2}}\\ L_{sa}=\sqrt{{\left(x_{a}-x_{s}\right)}^{2}+{\left(y_{s}-y_{a}\right)}^{2}} \end{array}$$

where *L_sd_*, *L_da_*, and *L_sa_* denote the flight distance of ultrasonic waves, which can be calculated through a two-dimensional coordinate position. *V*_1_ is the group velocity of the source in polymer, and *V*_2_ is the group velocity of the damage-scattered waves.

In the above, Δt is obtained by the detection electrode. Combined with known parameters such as *V*_1_, *V*_2_, electrode coordinates (*x_a_*, *y_a_*), and source coordinates (*x_s_*, *y_s_*), all possible locations of the damaged area can be obtained like an elliptical as shown in Figure 1e.

**Figure 2:**
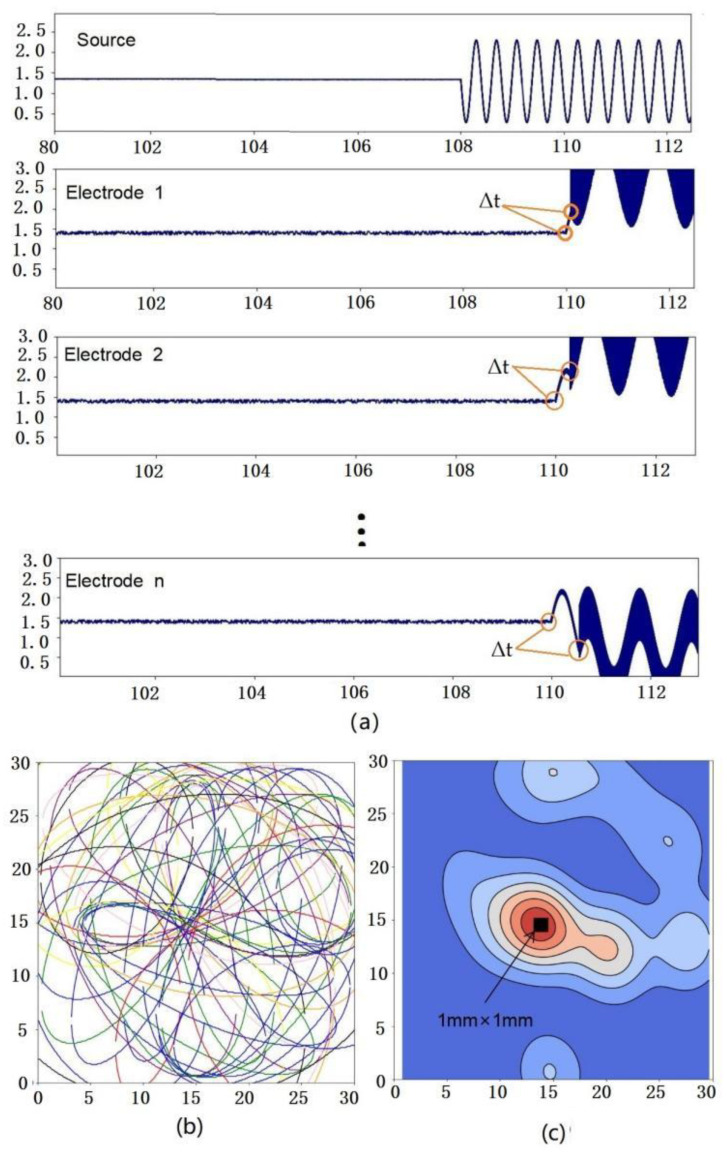
(**a**) The flight time of excitation wave obtained by electrodes; (**b**) Superposition diagram of multiple elliptical regions with damage location; (**c**) Cloud image of damage location measurement.

Meanwhile, to verify the feasibility of the detection method, a detection device with 4 × 4 array electrodes and 4 excitation sources is used to measure 30 mm × 30 mm graphene composite polymer films with 1 mm × 1 mm internal defects. The flight time Δt of the excitation wave is obtained by different electrodes as shown in Figure 2a. Then, multiple elliptical regions can be obtained using triangulation by 4 × 4 array electrodes as shown in Figure 2b, and the damage location information can be obtained by combining the kernel density algorithm with the weight analysis function as shown in Figure 2c. It can be seen that the red area is the measurement result of the damage location. Compared with the black real damage area, there is a large deviation in the shape and position of the measurement results. Because the location accuracy of the internal damage of the film is determined by the number of electrodes, the self-inductance of the film to the wave, and the location algorithm, to obtain high-precision detection results, it is necessary to increase the number of electrodes and optimize the detection algorithm.

## 3. Optimization of the Detection System and Location Algorithm

### 3.1. Detection Electrode Optimization

For the transducer of the sensor, the smaller the electrode gap, the higher the sensitivity to the resistance change. The interdigital electrode transducer prepared by a microelectronic process with ultra-high detection sensitivity is usually used to measure resistance change. The defect self-monitoring system of the conductive polymer film is based on the self-inductance of the 2-D graphene nanocomposites to ultrasonic wave, the higher the sensitivity of detecting resistance variation, the higher the precision of time-of-flight extraction, and the smaller the error of damage location. In addition, the health monitoring of sensing film during the working process of the sensor is of great significance to the study of the sensitivity mechanism (the change of electrode resistance can reflect the tunneling effect between graphene particles) and reliability (whether internal damage of sensitive membrane is induced after contacting the detection target) of the sensor.

**Figure 3:**
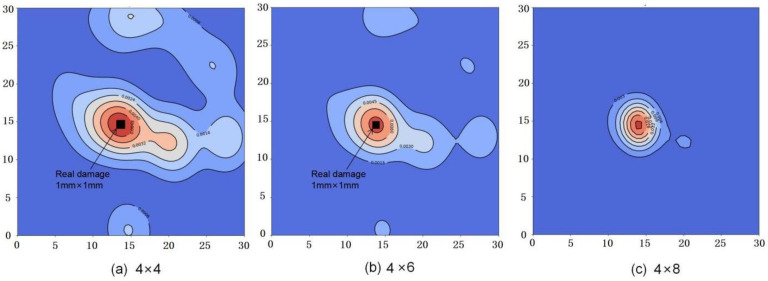
Cloud image of damage location measurement with (**a**) 4 × 4 electrodes; (**b**) 4 × 6 electrodes; (**c**) 4 × 8 electrodes.

Theoretically, the more electrode pairs, the higher the spatial damage detection resolution, but the more complex the signal processing circuit, the longer the response time. Combined with the positioning algorithm and response time, the design parameters of the transducer including the electrode gap and the number of electrodes are optimized to improve the positioning accuracy of damage in graphene nanocomposites. Using the same algorithm, detection devices with 4 × 6 and 4 × 8 array electrodes are used to measure 30 mm × 30 mm graphene composite polymer films with 1 mm × 1 mm internal defects, respectively. The red area of the cloud image is the measurement result of the damage location with a different number of electrodes as shown in Figure 3. It can be seen that the measurement error is greatly reduced with the increase in the number of detection electrodes, and the measured results almost coincide with the real damage area as shown in Figure 3c. The damage measurement error of devices with 4 × 8 pairs of electrodes for 30 mm × 30 mm graphene composite polymer is less than 2%. Meanwhile, the measurement error and response time of the detection system with different electrodes are shown in Table 1.

### 3.2. Algorithm Optimization

Each electrode generates an elliptical region containing the damage, and the two ellipses generate multiple intersections including real intersections and redundant intersections. To eliminate redundant intersection points and determine the location of the real intersection points, the common method is to add weights to the elliptic path to effectively reduce the redundant points, such as the probability-based diagnostic imaging (PDI) algorithm [14,15]. In the experiment, there are many misjudgments with this algorithm because there may be multiple dense point sets. The kernel density estimation algorithm can effectively solve the problem of misjudgment, but it needs a large sample size.

**Figure 4:**
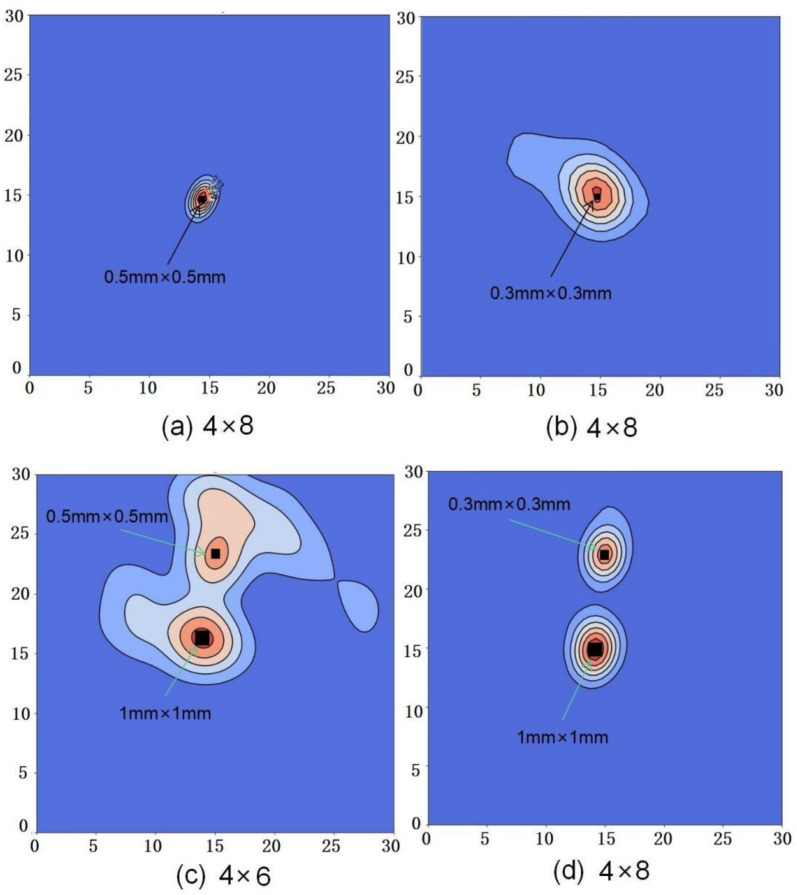
Cloud images of a single damage area with (**a**) 0.5 mm × 0.5 mm and (**b**) 0.3 mm × 0.3 mm; Cloud images of multiple damages with the different areas (**c**) 1 mm × 1 mm and 0.5 mm × 0.5 mm; and (**d**) 1 mm × 1 mm and 0.3 mm × 0.3 mm.

To eliminate the interference of dense intersections near the excitation source, only the signals of three groups of electrodes directly opposite the left and right of the excitation source are analyzed and processed. The detection diagrams of a single damaged area are shown in Figure 4a,b, respectively. The relative error of a single damage area with 0.5 mm × 0.5 mm and 0.3 mm × 0.3 mm is 5% and 60% respectively. The number of electrodes can be increased to improve the damage resolution and reduce the positioning error, but it will increase the response time and computing power cost.

In addition, this paper also realizes multiple damage detection. As shown in Figure 4c,d, multiple damage detection is realized, and the relative error of damage area with 1 mm × 1 mm is less than 5%. It also can be seen that the positioning error for smaller sizes is large, such as the relative error of damage area with 0.5 mm × 0.5 mm is close to 100 % in Figure 4c. By increasing the number of electrodes, the position error is reduced to 30% for 0.3 mm × 0.3 mm as shown in Figure 4d.

Due to the different times of multiple damage scattering waves reaching the same electrode, different flight time intervals can be obtained theoretically. However, it is difficult to extract the arrival time of more than twice the scattered waves by current methods because the scattered waves will produce a lot of noise after the superposition of electrodes. In the experiment of this paper, the first moment of the scattered wave arrives at the electrode is extracted, and the time of the others scattered wave with a longer path arrives is discarded. Compared with single damage detection, the increase of the number of damage locations reduces the number of elliptical areas at a single damage location, which can be seen as a disguised reduction in the number of electrodes, thus reducing the detection accuracy of damage.

In our future work, the multiple damage location accuracy can be further improved by optimizing the time-of-flight acquisition method based on noise control or filtering methods to accurately extract the arrival time of multiply scattered waves at the same electrode.

## 4. Conclusions

This paper developed a defective self-monitoring device of the conductive polymer film based on the self-inductance of the 2-D graphene nanocomposites to ultrasonic waves. This method can be applied to a variety of conductive polymer composite films, such as epoxy resin, polyimide, polyethylene, polyurethane, glass fiber, silicone rubber, polyaniline, etc., which can be nano-engineered and endowed with the capability of self-perceiving guided ultrasonic waves. As elastic waves traverse graphene nanocomposites, the wave-induced strain modulates the inter-distance among nanoparticles and consequently triggers the quantum tunneling effect, which alternates the measured resistivity of the polymer. Aiming at the sensor application field, the health monitoring of sensing film during the working process of the sensor is of great significance to the study of the sensitivity mechanism because the change of electrode resistance can reflect the tunneling effect between graphene particles. Therefore, the transducer with an integrated electrode array and excitation source for sensors are optimized. The results show that the measurement error of devices with 4 × 8 pairs of electrodes for 1 mm × 1 mm single damage in 30 mm × 30 mm graphene composite polymer is less than 2%, and the lower detection limits of damage size are 0.3 mm × 0.3 mm. In addition, multiple dafmage detection is also realized, the relative error of damage area with 1 mm × 1 mm is less than 5%, and the position error is reduced from 60% to 30% for 0.3 mm × 0.3 mm by optimizing the detection algorithm. The location accuracy of the internal damage of the film is determined by the number of electrodes and the time of flight acquisition of scattered waves, to obtain high-precision detection results, it is necessary to increase the number of electrodes and optimize the time of flight acquisition method. More than that, the devices are also used to detect the structural health of thin films as an electromagnetic wave absorbing layer and anti-corrosion protective coating layer.

## Figures and Tables

**Table 1:** The measurement error and response time of the detection system with different electrodes.

Electrodes Array	Measurement Error	Response Time
4 × 4	100%	15
4 × 6	40%	24
4 × 8	2%	30

## Data Availability

Data are contained within the article.
